# Repertoires of the Nucleosome-Positioning Dinucleotides

**DOI:** 10.1371/journal.pone.0007654

**Published:** 2009-11-02

**Authors:** Thomas Bettecken, Edward N. Trifonov

**Affiliations:** 1 CAGT – Center for Applied Genotyping, Max Planck Institute of Psychiatry, Munich, Germany; 2 Genome Diversity Center, Institute of Evolution, University of Haifa, Haifa, Israel; 3 Division of Functional Genomics and Proteomics, Faculty of Science, Masaryk University, Brno, Czech Republic; National University of Ireland Galway, Ireland

## Abstract

It is generally accepted that the organization of eukaryotic DNA into chromatin is strongly governed by a code inherent in the genomic DNA sequence. This code, as well as other codes, is superposed on the triplets coding for amino acids. The history of the chromatin code started three decades ago with the discovery of the periodic appearance of certain dinucleotides, with AA/TT and RR/YY giving the strongest signals, all with a period of 10.4 bases. Every base-pair stack in the DNA duplex has specific deformation properties, thus favoring DNA bending in a specific direction. The appearance of the corresponding dinucleotide at the distance 10.4 xn bases will facilitate DNA bending in that direction, which corresponds to the minimum energy of DNA folding in the nucleosome. We have analyzed the periodic appearances of all 16 dinucleotides in the genomes of thirteen different eukaryotic organisms. Our data show that a large variety of dinucleotides (if not all) are, apparently, contributing to the nucleosome positioning code. The choice of the periodical dinucleotides differs considerably from one organism to another. Among other 10.4 base periodicities, a strong and very regular 10.4 base signal was observed for CG dinucleotides in the genome of the honey bee *A. mellifera*. Also, the dinucleotide CG appears as the only periodical component in the human genome. This observation seems especially relevant since CpG methylation is well known to modulate chromatin packing and regularity. Thus, the selection of the dinucleotides contributing to the chromatin code is species specific, and may differ from region to region, depending on the sequence context.

## Introduction

A number of different dinucleotides have been indicated to be involved in the nucleosome positioning sequence pattern [1]–[5]. However, only for the dinucleotides AA, TT, RR and YY a prominent periodical appearance in natural chromatin DNA sequences could be demonstrated directly by positional autocorrelation (distance) analysis [1], [6]–[7]. These signal dinucleotides are preferentially appearing at distances which are multiples of the nucleosome DNA structural period, 10.4±0.2 bases. This value has been estimated by several independent approaches – from beat effect analysis of DNaseI digestion data [8], from superhelicity of the nucleosome DNA [9], from data on digestion by various nucleases (for review see reference [10]), and most recently - from analysis of DNA sequence periodicity, and from known coordinates of phosphates in the crystallized nucleosomes [11]. The 10.4 base periodicity of dinucleotides today is an undebated hallmark of nucleosome positioning. There are certain phase relationships between various dinucleotides, reflecting preferential orientations of the respective base-pair stacks, this way facilitating unidirectional bending of the nucleosome DNA. These phase relationships can be expressed in form of matrices of bendability [12], [2], [5], where, according to the recent data of Gabdank et al. [5], the highest positional selectivity is displayed by the dinucleotides AT and CG. Participation of CG dinucleotides in the nucleosome positioning has been demonstrated experimentally [13]–[15] and implicated from computational analysis of the Alu sequence repeats [16].

In this work we applied the distance analysis technique for determining which of the 16 dinucleotides display the 10.4 base periodicity in thirteen diverse eukaryotic organisms for which the complete, or at least nearly complete, genome sequences are available.

## Results

**Figure 1:**
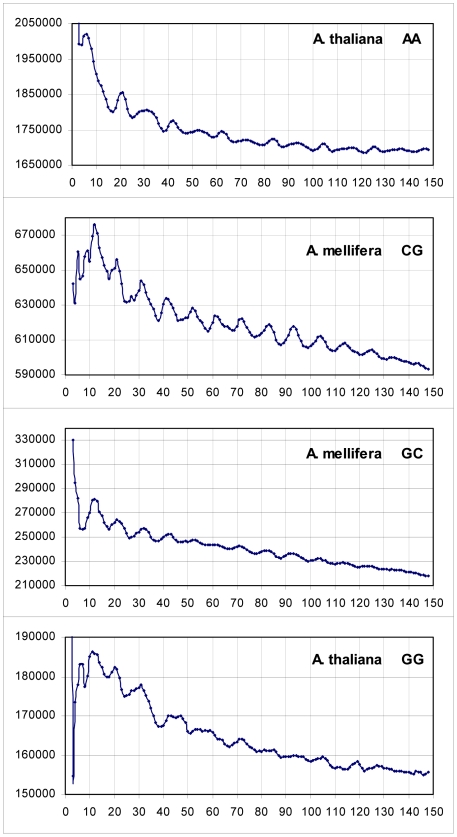
The four most prominent and clear dinucleotide periodicities amongst all thirteen genomes and dinucleotides analysed. The counts in the histograms are smoothened by averaging over 3 bases. For each genome the total counts (Y axis) summed over all chromosomes entering the study are plotted as function of the distances (X axis) in the interval 1–150 bases.

A total of 208 periodicity plots for 13 eukaryotic genomes and all 16 dinucleotides are calculated, revealing that each one of the 16 dinucleotides clearly shows the periodical positioning in at least one of the genomes analyzed. In Fig. 1 the most prominent examples of emerging periodicities are shown, as calculated from the genomes of *A. thaliana* (AA and GG) and *A. mellifera* (CG and GC). All histograms display the maxima at positions closely corresponding to multiples of 10.4 bases, all the way to 104 bases and even beyond. This appears especially clear in the case of CG in *A. mellifera*. Here the maxima are observed at positions that are the closest integers to the 10.4 xn series: 21(20.8), 31(31.2), 41(41.6), 52(52.0), 72(72.8), 83(83.2), 93(93.6), 104(104.0), 114(114.4), 125(124.8). In the other three graphs the fit is almost as good. Because of some reason, probably due to various noise components of the distance histograms, the 1^st^ peaks in Fig. 1 appear rather at positions 11 or 12, up to 1.6 bases off the expected 10.4 base position.

**Figure 2:**
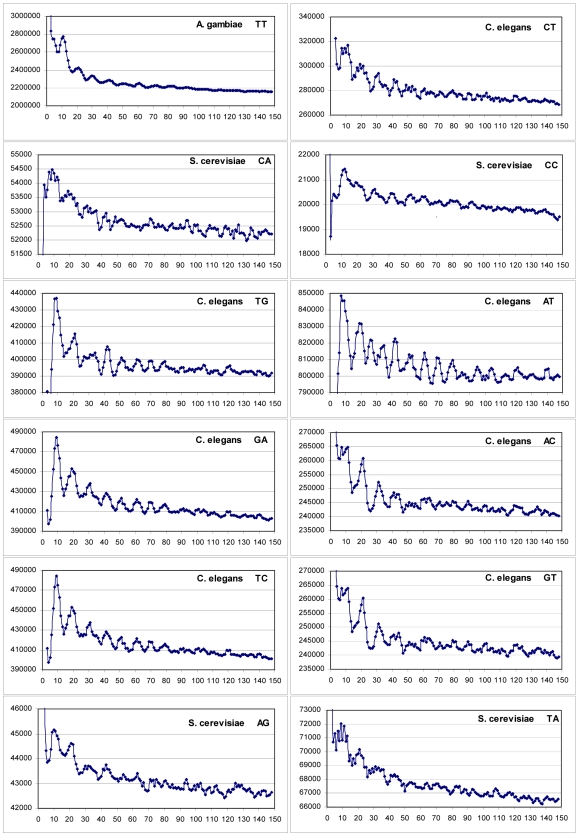
Other examples of periodic appearance of various dinucleotides in an interval up to 150 bases. Graphs were chosen to represent the best visible signal for each of the remaining 12 dinucleotides, in addition to the ones in Fig. 1. Otherwise, data is arranged the same way as in Fig. 1.

Other clear examples of the easily visible periodicities, for the remaining 12 dinucleotides are presented in Fig. 2, where three or more peaks of the 10.4 xn series can be seen in each case. As in Fig. 1, the most representative curves are selected for each dinucleotide. Of the twelve plots, the lowest amplitude oscillations are observed for dinucleotides of *S. cerevisiae* (CA, CC, AG and TA). However, respective excess values over background in these cases all exceed 2.9 STD (see Methods), which is in full agreement with the estimates first made by Cohanim et al. [6]. The first maxima in the examples shown are observed typically at position 10±1. The precise positioning of most of the peaks observed in Fig. 1 and 2 identifies the 10.4 base repeat of nucleosome DNA. It is clearly distinct from sequence periods 10.0 and 11.0 bases, characteristic for Archaea and Eubacteria, respectively [17], and from the structural period 10.55 bases for free DNA (e.g. in [10]).

The figures also demonstrate that each one of the 16 dinucleotides may contribute to the overall 10.4 base periodicity, though not in every genome and not to the same degree. In Table 1 the most prominent periodic dinucleotides of the thirteen species are marked by a “+”. At least three clear peaks of the 10.4 xn series are present in all respective plots for the positive cases. The periodical AA and TT dinucleotides dominate (clearly visible in 9 genomes of 13). CG and GC are the next most popular ones (6 genomes). In human sequences, the CG dinucleotide is the only recognizable periodical component (see Table 1), which is observed for the first time, in this work. The least prominent periodicity is displayed by the dinucleotides AC, GT (in *C. elegans* only) and TA (in *S. cerevisiae* only) as shown in Fig. 2 and Table 1. At the same time, these two genomes display the largest repertoires of periodical dinucleotides. This confirms the earlier result obtained by Cohanim et al. for yeast [6]. The mouse genome does not show any obvious periodicity, neither in unfiltered nor in repeat-filtered sequence. Some weak oscillations may be detected, perhaps, by more sophisticated analysis.

**Table 1:** Dinucleotides displaying a clear 10.4 base periodicity in the set of thirteen eukaryotic genomes.

	AA	TT	CG	GC	CA	TG	AG	CT	AT	GG	CC	GA	TC	AC	GT	TA
*S. cerevisiae*	+	+	+	+	+	+	+	+	+	+	+	+	+	−	−	+
*C. elegans*	+	+	+	+	+	+	+	+	+	−	−	+	+	+	+	−
*A. thaliana*	+	+	−	+	+	+	−	−	+	+	−	−	−	−	−	−
*D. rerio*	+	+	−	+	−	−	−	−	−	+	+	−	−	−	−	−
*C. albicans*	+	+	−	−	+	+	−	−	−	−	−	−	−	−	−	−
*D. melanogaster*	+	+	+	+	−	−	−	−	−	−	−	−	−	−	−	−
*A. mellifera*	+	+	+	+	−	−	−	−	−	−	−	−	−	−	−	−
*A. gambiae*	+	+	−	−	−	−	−	−	−	−	−	−	−	−	−	−
*C. reinhardtii*	+	+	−	−	−	−	−	−	−	−	−	−	−	−	−	−
*G. gallus*	−	−	−	−	−	−	+	+	−	−	−	−	−	−	−	−
*D. discoideum*	−	−	+	−	−	−	−	−	−	−	−	−	−	−	−	−
*H. sapiens*	−	−	+	−	−	−	−	−	−	−	−	−	−	−	−	−
*M. musculus*	−	−	−	−	−	−	−	−	−	−	−	−	−	−	−	−

## Discussion

From our calculations it became evident, that each one of the 16 dinucleotides shows the 10.4 bp periodical positioning in several or at least one of the genomes analyzed. However, not every genome displays a periodicity, the mouse genome being such an exception. According to Table 1, there seems to be no visible correlation of sizes of dinucleotide repertoires with taxonomy. We demonstrate for the first time that CG dinucleotides show a strong positional periodicity, best seen in the CG-rich genome of the honey bee *A. mellifera* and in *D. melanogaster* (data not shown). The observed oscillations follow the nucleosome DNA period of 10.4 bases. This confirms in the most straightforward way the participation of the CG dinucleotides together with other elements in the formation of the 10.4 base periodical nucleosome DNA sequence pattern. Surprisingly, when analyzing the human genome in the same way, a clear periodicity of dinucleotides is visible exclusively for the dinucleotide CG. AA/TT dinucleotide positions come out periodical in nine of thirteen genomes tested. The warm blooded vertebrates *G. gallus, H. sapiens, M. musculus* and the amoeba *D. discoideum* make a notable exception here. It has been reported earlier [18]–[19] that human nucleosome DNA sequences do not display AA/TT periodicity. Rather, RR/YY dinucleotides appear periodically in the nucleosome DNA. However, our analysis of the human genomic sequences shows lack of the RR/YY periodicity. The CG periodicity in human sequences has become evident now for the first time. Together with the spectacular example of the *A. mellifera* genome, where CG dinucleotides are 1.7 times more frequent than the genomic base composition would suggest [20], the CG signal in the human genome (with CGs considerably underrepresented) confirms the role which these dinucleotides, apparently, play in the nucleosome positioning. Participation of CG in the positioning is of special value because of the duality of the CG dinucleotides, in which the cytosines can be either methylated or non-methylated. The nucleosomes formed on CG containing sequences may well have an “epigenetic” property [16], their stability and positions being modulated by the CG methylation, this way possibly influencing the expression level of genes located nearby.

The “weakest” dinucleotides in terms of periodicity are AC, GT and TA. This may or may not mean that, actually, the periodicity in these cases is just due to passive sequence exclusion effects caused by strong periodicities of other dinucleotides [5]. Indeed, the *S. cerevisiae* genome shows strong periodicities of other dinucleotides. It is also quite possible, that TA elements have deformational properties very much suitable for nucleosome positioning. In strong nucleosome forming DNA fragments extracted from a pool of synthetic random sequences, TA, indeed, is frequent and displays a clear periodicity [21]. However, since TA steps are characterized by lowest stability [22] it remains open as yet whether such sequences with periodical potential kinks at TA may reside in natural nucleosomes as well.

Our calculation results (Table 1) show that in every genome a different set of periodical dinucleotides is prominent. Accordingly, one would expect that a number of different nucleosome positioning dinucleotide repertoires exist. Each one of them may appear as the dominant one at the whole-genome scale, depending on the sequence composition of the organism. With this in mind, it seems very reasonable to propose that different genomic regions may well harbor different nucleosome positioning repertoires, depending on several factors. These could be the dinucleotide frequencies, the G+C content [23], the presence and type of repeating sequences which may attract strong nucleosomes or impose their sequence structure on the positioning signal, and possibly also some other species-specific sequence biases.

The eukaryotic genome sequences are massively involved in nucleosomes, in protein-coding and even more in non-coding sequences. The richness of the dinucleotide repertoires observed strongly points to direct structural aspects of single nucleotide polymorphisms, SNPs, and SNP haplotypes, with all their functional implications. Nucleosome positioning studies may well have the potential to help in interpretation of genetic association studies results, when associations are found with SNPs mapping to “gene deserts” [24].

## Methods

For this overview of dinucleotide periodicities in eukaryotes, we selected sequences from a number of well characterized model organisms, supplemented by others in order to be more representative. Besides, we only included genomes, where a documented assembly into chromosomes was available. Genomic sequences of *Caenorhabditis elegans* (ce6, genome.ucsc.edu), *Arabidopsis thaliana* (build of 20 Dec 2007, ftp.arabidopsis.org), *Anopheles gambiae* (AgamP3, agambiae.vectorbase.org), *Apis mellifera* (apiMel4, ftp.hgsc.bcm.tmc.edu), *Saccharomyces cerevisiae* (sacCer1, genome.ucsc.edu), *Gallus gallus* (galGal3, genome.ucsc.edu), *Mus musculus* (mm9, genome.ucsc.edu), *Homo sapiens* (hg18, genome.ucsc.edu), *Candida albicans* (Ca21, www.candidagenome.org), *Chlamydomonas reinhardtii* (Chlre4, genomes.jpg-psf.org), *Danio rerio* (danRer5, genome.ucsc.edu), *Dictyostelium discoideum* (Ver. May 2009, dictybase.org) and *Drosophila melanogaster* (dm3, genome.ucsc.org) were downloaded from the respective server. Dinucleotide positions in sequences (grouped into chromosomes) were determined by pattern search and recorded in a file. From these files, for every dinucleotide the distances to the next identical dinucleotides in a downstream interval of 150 bases were calculated (positional autocorrelation analysis) and recorded. Then, the start point for the next interval was shifted to the next identical dinucleotide. Calculated distances were summed over the chromosomes (*C. elegans* chrI to chrV and chrX, *A. thaliana* chr1 to chr5, *A. gambiae* chr2L, chr2R, chr3L, chr3R, *A. mellifera* group1 to group16, *S. cerevisiae* chr1 to chr16, *G. gallus* chr1 to chr28, *M. musculus* chr1 to chr19, *H. sapiens* chr1 to chr22, *C. albicans* chr1 to chr5, *C. reinhardtii* Chr1 to Chr17, *D. rerio* Chr1 to Chr25, *D. dictyostelium* Chr1 to chr6 and *D. melanogaster* chr2L, chr2R, chr3L, chr3R and chr4). Counts for distances were arranged in histograms and smoothened by averaging over 3 bases. Statistical significance of the data has been estimated as in [1] by relating excess values in the observed periodical peaks to respective background scores. In case of the weakest oscillations found, for *S. cerevisiae* (CA, CC, AG and TA, see Fig. 2), the cumulative effects of the excess values amount, respectively, to 3.0, 2.9, 2.9 and 3.9 STD. This is in full agreement with the estimates first made in [6]. All 208 plots were evaluated for visibility of a 10.4 bp periodicity. When visible, a “+” was entered into Table 1, otherwise a “−”. The data in the rows and columns of Table 1 are sorted by the size of the repertoires of periodic dinucleotides. Human sequences have been analysed both as unfiltered and after filtering out major repeats, Alu repeats in particular, by using the sequence data available under the label “masked” (hg18, file chromFaMasked.zip, genome.ucsc.edu). The data from the filtered sequence was considered for the *H. sapiens* entry in Table 1. Mitochondrial and unmapped sequences were not taken for the analyses.
